# The RNA-Binding Protein KSRP Modulates Cytokine Expression of CD4^+^ T Cells

**DOI:** 10.1155/2019/4726532

**Published:** 2019-08-14

**Authors:** Rudolf Käfer, Lisa Schmidtke, Katharina Schrick, Evelyn Montermann, Matthias Bros, Hartmut Kleinert, Andrea Pautz

**Affiliations:** ^1^Department of Pharmacology, University Medical Center of Johannes Gutenberg University, Mainz, Germany; ^2^Department of Dermatology, University Medical Center of Johannes Gutenberg University, Mainz, Germany

## Abstract

The KH-type splicing regulatory protein (KSRP) is a RNA-binding protein, which regulates the stability of many mRNAs encoding immune-relevant proteins. As KSRP regulates innate immune responses, for instance by the modulation of type I interferon mRNA stability, we were interested whether knockdown of the protein (KSRP^−/−^) interferes with T cell activation and polarization. Polyclonally stimulated KSRP^−/−^ CD4^+^ T cells proliferated at a higher extent and higher frequency and expressed the activation marker CD25 more than wild-type T cells. In supernatants of stimulated KSRP^−/−^ CD4^+^ T cells, levels of IL-5, IL-9, IL-10, and IL-13 were observed to be increased compared to those of the control group. KSRP^−/−^ CD8^+^ T cells showed no altered proliferative capacity upon polyclonal stimulation, but supernatants contained lower levels of interferon-*γ*. Similar changes in the cytokine expression patterns were also detected in T cells derived from KSRP^−/−^ mice undergoing arthritis induction indicative of a pathophysiological role of KSRP-dependent T cell polarization. We demonstrated the direct binding of KSRP to the 3′ untranslated region of IL-13, IL-10, and IFN-*γ* mRNA in in vitro experiments. Moreover, since IL-4 mRNA decay was reduced in KSRP^−/−^ CD4^+^ T cells, we identify KSRP as a negative regulator of IL-4 expression. These data indicate that overexpression of IL-4, which constitutes the primary inducer of Th2 polarization, may cause the Th2 bias of polyclonally stimulated KSRP^−/−^ CD4^+^ T cells. This is the first report demonstrating that KSRP is involved in the regulation of T cell responses. We present strong evidence that T cells derived from KSRP^−/−^ mice favor Th2-driven immune responses.

## 1. Introduction

The immune system is composed of innate (dendritic cells, macrophages, granulocytes, etc.) and adaptive (T and B cells) immune cell types. To coordinate an effective immune response to pathogens, an extensive cross-talk between both systems is required. The communication between different immune cells is mediated in part by cytokines released from cells in response to different stimuli. These cytokines have pleiotropic functions, including the regulation of immune cell differentiation and activation [1]. Therefore, it is obvious that dysregulation of cytokine expression is important for the pathogenesis of many diseases, for example, for chronic inflammatory autoimmune diseases such as rheumatoid arthritis [2].

In adaptive immune responses, the cytokine environment is important for the activation and differentiation of CD4^+^ T cells into distinct effector T helper (Th) cell subsets (Th1, Th2, Th9, or Th17). Each Th cell subset is characterized by a predominant cytokine pattern, which determines the function of the cell in immune responses. Th1 cells typically produce IFN-*γ* and mediate cellular immune responses, whereas Th2 cells are characterized by IL-4, IL-5, IL-10, and IL-13 secretion and are essential for humoral immune defense mechanisms [3].

Tight regulation of cytokine expression is necessary to avoid an overwhelming and destructive immune response. Cytokine expression is regulated by transcriptional, posttranscriptional, and posttranslational mechanisms. Whereas transcription factors and epigenetic mechanisms are responsible for transcriptional control, posttranscriptional regulation (splicing, mRNA editing, stability, translatability, and localization) often depends on RNA-binding proteins (RBP) [4]. These proteins either stabilize (HuR) or destabilize (AUF1, tristetraprolin, and KSRP) cytokine mRNAs by binding to specific sequence elements, often AU-rich elements (ARE) located in the 3′ untranslated region (3′-UTR) of the mRNA. The importance of RBPs like AUF1, tristetraprolin (TTP), or KSRP (KH-type splicing regulatory protein, also named KHSRP or far upstream sequence-binding protein 2 (FuBP2)) for immune regulatory processes has been demonstrated in knockout animals [5–7].

KSRP is a multifunctional, single-stranded nucleic acid- (DNA- or RNA-) binding protein. KSRP has been described to regulate c-myc transcription by binding to the far upstream sequence of the myc promoter [8] and also to regulate TNF-*α* promoter activity [9]. Additionally, KSRP is involved in different posttranscriptional processes, such as regulation of mRNA splicing, stability, and translatability and microRNA (miRNA) maturation. The protein binds to AREs in the 3′-UTR of mRNAs and recruits enzymes involved in the 5′- and 3′-mRNA decay [10]. Therefore, it negatively regulates the expression of immune modulatory cytokines like TNF-*α* and type I interferons [7, 11]. It is likely that KSRP modulates cytokine production also via its ability to promote the maturation of a specific subset of miRNAs [12]. Here, KSRP binds to the terminal loop of the target miRNA precursors and thereby promotes their maturation.

An important role of KSRP in the regulation of innate immune responses, especially in antiviral signaling, has been demonstrated. The activation of the retinoic acid-inducible gene I (RIG-I) receptor, which triggers a signaling cascade that is important for antiviral defense mechanisms, is inhibited by KSRP. Therefore, in the absence of KSRP protein viral replication is reduced, due to enhanced RIG-I signaling [13]. Moreover, KSRP has been described as a direct negative regulator of type I IFN mRNA stability. Thus, knockdown of the KSRP gene in mice (KSRP^−/−^ mice) resulted in increased type I IFN expression and subsequently led to an enhanced herpes simplex virus 1 resistance [7].

KSRP is proposed to be an important negative regulator of proinflammatory gene expression [11]. Therefore, we expected that knockdown of this protein would enhance the expression of proinflammatory factors and aggravate inflammatory immune responses and diseases such as rheumatoid arthritis in mice. Upon induction of collagen antibody-induced arthritis (CAIA), a well-established arthritis model, in C57BL/6 KSRP^−/−^ mice, the opposite effect occurred: the KSRP^−/−^ animals were protected against CAIA [14]. The results from the CAIA model indicate a much more complex role of KSRP in the regulation of immune responses *in vivo* as expected. Further, it has been demonstrated that mice deficient in B and T cells develop a more severe CAIA disease than control mice. This suggests that B and T cells have a disease-modifying role in this model [15] and raised our interest in the role of KSRP for T cell function under physiological and pathophysiological conditions.

## 2. Materials and Methods

### 2.1. Materials

Trypsin, glutamine, sodium pyruvate, and all oligonucleotides were purchased from Sigma-Aldrich, Deisenhofen, Germany. All cell-culture-grade plastic materials were obtained from Greiner, Solingen, Germany. FCS and DMEM were purchased from PAN-Systems, Nürnberg, Germany. Restriction enzymes, polynucleotide kinase (PNK), calf intestinal alkaline phosphatase (CIAP), Taq DNA polymerase, Klenow DNA polymerase, dNTPs, and NTPs were purchased from New England Biolabs, Frankfurt a.M., Germany. T7 and T3 RNA and DNase I polymerase were obtained from Roche Diagnostics, Mannheim, Germany. pCR-Script was obtained from Agilent Technologies, Waldbronn, Germany. The plasmids pGL3control_TNF3UTR and pGL3control_TNF3UTRAUdel were kind gifts from Dr. W.F.C. Rigby [16].

### 2.2. Animals

All mice were housed in accordance with standard animal care requirements and were maintained under specified pathogen-free conditions on a 12/12-hour light/dark cycle. Water and food were given *ad libitum*. The animal studies were approved by the ethical board and were performed in accordance with the German animal protection law and the guidelines for the use of experimental animals as stipulated by the Guide of Care and Use of Laboratory Animals of the National Institutes of Health. Mice were euthanized by *i.p.* injection of 1% pentobarbital in PBS.

KSRP^+/-^ mice [7] had a C57BL/6 background. Experimental KSRP^−/−^ and KSRP^+/+^ (WT) animals were obtained by mating KSRP^+/-^ animals. Genotyping of the animals was performed by polymerase chain reaction, using primers that span the region of the wild-type KSRP gene flanked by loxP sites which were deleted by the Cre recombinase [7, 14]. The following oligonucleotides were used for genotyping the KSRP-locus: KSRP-wt-for GCGGGGAGAATGTGAAGG, KSRP-ko-for CTCCGCCTCCTCAGCTTG, and KSRP-wt/ko-rev GAGGCCCCTGGTTGAAGG.

### 2.3. Immunomagnetic T Cell Isolation

Spleen cells derived from WT and KSRP^−/−^ mice were used for immunomagnetic isolation of untouched CD4^+^ and CD8^+^ T cells by negative selection as recommended by the manufacturer (Miltenyi Biotec GmbH, Bergisch Gladbach, Germany).

### 2.4. T Cell Proliferation Assays

Untouched CD4^+^ and CD8^+^ T cells derived from naïve mice or nylon wool-enriched T cells [17] were resuspended in medium (IMDM supplemented with 5% FCS (PAA, Cölbe, Germany), 2 mM L-glutamine, 100 U/ml penicillin, 100 *μ*g/ml streptomycin (all from Sigma-Aldrich, Deisenhofen, Germany), and 50 *μ*M *β*-mercaptoethanol (Carl Roth, Karlsruhe, Germany)) at a density of 5 × 10^4^/100 *μ*l and were seeded on 96-well tissue-culture plates (Corning, Cambridge, MA). In some experiments, T cells were serially diluted in triplicates. T cell populations were polyclonally stimulated with plate-bound anti-CD3- (1 *μ*g/ml) and anti-CD28- (2 *μ*g/ml) specific antibodies (both from Affymetrix/eBioscience, San Diego, CA) for 96 h. In order to assess the proliferation of T cells, ^3^H thymidine (0.5 *μ*Ci/well) was applied for the last 16-18 h of the incubation time. Then, cells were harvested onto glass fiber filters, and retained radioactivity was measured in a liquid scintillation counter (1450 MicroBeta TriLux, LKB Wallac, Turku, Finland).

### 2.5. Flow Cytometry

For subsequent flow cytometric analysis, T cell populations (5 × 10^6^/ml) (see above) were seeded into wells (1 ml) of 24-well culture plates and were polyclonally stimulated. After 96 h, cells were washed in PBS with 2% FCS, and Fc receptor-mediated binding of antibodies was blocked by incubation with rat anti-mouse CD16/CD32 mAb (supernatant of B cell hybridoma 2.4G2) for 10 min at 4°C. All cells were incubated for 20 min at 4°C with PE-anti-CD3, APC-anti-CD4, eFluor450-anti-CD8 mAbs (all from Affymetrix/eBioscience/Thermo Fisher Scientific, Darmstadt, Germany), and FITC-anti-CD25 mAb purchased from BD Biosciences Pharmingen (Heidelberg, Germany). After washing, samples were fixed with 0.7% paraformaldehyde (in PBS). Cells were analyzed using a FACSCanto II (BD Biosciences, Heidelberg, Germany) flow cytometer equipped with BD DIVA Software. Data were analyzed using FlowJo software (FlowJo, Ashland, USA).

### 2.6. Cytometric Bead Array Analyses

Supernatants of polyclonally stimulated T cell cultures (see above) were collected, and cytokine contents were determined by Cytometric Bead Array (CBA) [18] (BD Biosciences, Heidelberg, Germany). The CBA assays allowed quantification of cytokine concentrations between 10 pg/ml and 10 ng/ml based on serial cytokine dilutions tested in parallel as a standard. Samples were measured in a LSR II flow cytometer (BD Biosciences, Heidelberg, Germany). Data were analyzed using the FCAP Array Analysis Software (BD Biosciences, Heidelberg, Germany).

### 2.7. Induction of Collagen Antibody-Induced Arthritis

CAIA was induced in mice using a mixture of 5 collagen type II- (CII-) specific monoclonal antibodies (Arthrogen-CIA 5-clone cocktail kit, Amsbio, Frankfurt a.M. Germany) suspended in sterile PBS as described by the manufacturer and described before [14].

### 2.8. Cloning of the 3′-UTR Sequences of IL-5, IL-9, IL-10, and IFN-*γ* mRNA

To obtain constructs for the generation of RNA probes for pull-down experiments, the 3′-UTR sequences of the human IL-5, IL-9, IL-10, and IFN-*γ* mRNA were cloned into the EcoRV site of pCR-Script.

To obtain the plasmids pCR-TNF-3UTR and pCR-TNF-3UTRdelAURE, the plasmids pGL3control_TNF3UTR and pGL3control_hTNF3UTRAUdel (kind gifts from Dr. W.F.C. Rigby [16] were digested with XbaI and treated with Klenow enzyme. The blunt-ended fragments containing the 3′-UTR of the human TNF-*α* mRNA (with or without the AREs) were isolated and ligated to pCR-Script digested with EcoRV and treated with CIAP.

To obtain the plasmid pCR-Script-IL-2-3′-UTR, pCR-Script-IL-5-3′-UTR, pCR-Script-IL-9-3′-UTR, pCR-Script-IL-10-3′-UTR, and pCR-Script-IFN*γ*-3′-UTR, RT-PCR reactions with RNA isolated from DLD-1 cells were performed using the oligonucleotides described in Table 1 as primers of the reaction.

The PCR fragments were purified, treated with PNK, and ligated into pCR-Script restricted with EcoRV and treated with CIAP.

The relevant DNA sequences of all plasmids were determined using the dideoxy chain termination method (StarSEQ, Mainz, Germany).

### 2.9. Cell Culture

The murine EL4 lymphoma T cell line (ATCC® TIB-39™) was cultivated in Iscoves's medium with 5% inactivated fetal bovine serum (FCS), 1% L-glutamine 200 mM, 1% sodium pyruvate, 50 *μ*M *β*-mercaptoethanol, 100 U/ml penicillin, and 100 *μ*g/ml streptomycin.

### 2.10. Protein Extracts

To obtain protein extracts for a pull-down assay, EL4 lymphoma T cells were lysed in RIPA buffer (50 mM Tris-HCl (pH 7.4), 150 mM NaCl, 2 mM EDTA, 10% glycerol, 1% NP-40, 1x complete EDTA-free protease, and phosphatase inhibitor cocktail) using an ultrasonic homogenizer. Protein concentration was determined using the Protein Assay Dye Reagent (Bio-Rad, München, Germany) as described by the manufacturer.

### 2.11. In Vitro Transcription and Pull-Down Assay

To generate biotinylated RNA probes, 1 *μ*g of linearized pCR-TNF-3′-UTR, pCR-TNF-3′-UTRdelARE, pCR-Script-IL-2-3′-UTR, pCR-Script-IL-5-3′-UTR, pCR-Script-IL-9-3′-UTR, pCR-Script-IL-10-3′-UTR, pCR-Script-IL-13-3′-UTR, and pCR-Script-IFN*γ*-3′-UTR plasmid were in vitro transcribed using a biotin RNA labeling mix (Epicentre, Madison, USA) as described by the manufacturer. The reaction was conducted at 37°C for 2 h in a volume of 18 *μ*l. Digestion of the plasmids was performed using 2 *μ*l DNase I (Roche Diagnostics, Mannheim, Germany) for 20 min at 37°C.

Biotinylated RNA transcripts were incubated with 100 *μ*l buffered aqueous streptavidin-agarose from *Streptomyces avidinii* (Sigma-Aldrich, Darmstadt, Germany) for 6 h at 4°C in 100 *μ*l binding buffer (10 mM HEPES (pH 7.9), 3 mM MgCl_2_, 5 mM EDTA, 2 mM DTT, 5% glycerol, 0.5% NP-40, 3 mg/ml heparin, and 0.5 mg/ml baker's yeast transfer RNA) supplemented with 40 mM KCl. Protein extracts from EL4 cells were precleared by incubating 5 mg of cell extract with 50 *μ*l buffered aqueous streptavidin-agarose for 2 h at 4°C. Streptavidin-agarose with biotinylated RNA transcripts were pelleted for 5 min at 13000 rpm and incubated with precleared protein extract and 400 *μ*l binding buffer with 40 mM KCl overnight at 4°C. Streptavidin-agarose was washed five times in 100 *μ*l binding buffer with 40 mM KCl. Proteins were eluted by incubating with 100 *μ*l elution buffer (10 mM HEPES (pH 7.9), 3 mM MgCl_2_, 5 mM EDTA, 2 mM DTT, 0.2% glycerol, and 2 M KCl) for 30 min at 4°C.

To identify the eluted proteins, streptavidin-agarose was pelleted and the supernatant was separated on SDS-polyacrylamide gels and transferred to a nitrocellulose membrane by semidry electroblotting. For the detection of KSRP, a polyclonal anti-KSRP antibody (Abcam, ab229660) was used. The immunoreactive proteins on the blot were visualized by the enhanced chemiluminescence detection system (Thermo Fisher Scientific, Darmstadt, Germany).

### 2.12. qRT-PCR

To analyze mRNA expression, total RNA was prepared by homogenizing cells or tissue samples in GIT buffer [19]. RNA was isolated and specific gene expression quantified in a two-step real-time RT-PCR as previously described [20] with the oligonucleotides listed in Table 2.

All oligonucleotides were purchased from Sigma-Aldrich, Deisenhofen, Germany.

Specific mRNA expression was normalized to murine RNA-polymerase II (Pol2A), GAPDH, or 18S rRNA. To calculate the relative mRNA expression, the 2^(−ΔΔC(T))^ method [21] was used.

### 2.13. Statistics

Data represent means+SEM. Statistical differences were determined by factorial analysis of variance followed by “Tukey's” or “Dunnett's” multiple comparison test. In the case of two means, classical *t*-test analyses were used. To compare T cell proliferation, two-way Anova analysis followed by multiple comparisons tests was performed. All statistical analyses were performed using GraphPad Prism 6.0.

## 3. Results

### 3.1. Knockdown of KSRP Protein Results in Increased CD4^+^ T Cell Proliferation

The modulation of innate immune responses by KSRP has been demonstrated recently, but almost nothing is known about its role in immune cells of the adaptive immune system. Therefore, we analyzed the consequences of KSRP knockdown on T cell function. Knockdown of KSRP in T cells was confirmed by western blot experiments (Sup.  Figure 1). Total CD4^+^ and CD8^+^ T cell numbers in the spleen did not differ between KSRP knockout and wild-type mice and showed no difference in CD25 expression (Sup.  Table 1 and data not shown). These observations suggested that KSRP deficiency had no major effect on the T cell compartment.

**Figure 1:**
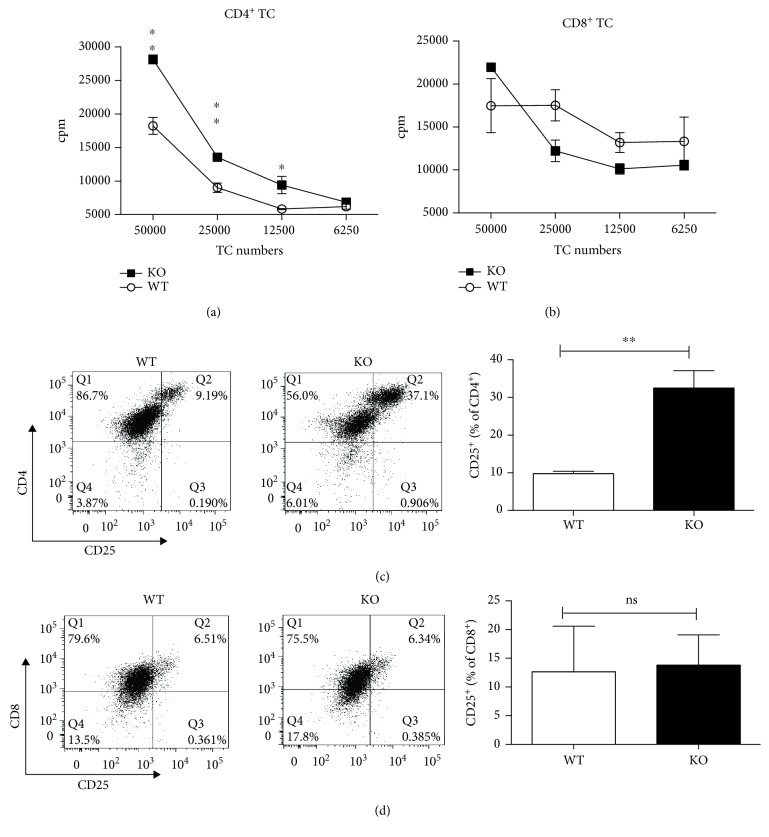
Inactivation of the KSRP gene enhances proliferation of polyclonally stimulated CD4^+^ T cells. Magnetic bead separation was used to isolate untouched CD4^+^ and CD8^+^ T cells from KSRP^+/+^ (WT) and KSRP^−/−^ (KO) mice. T cells were polyclonally stimulated with plate-bound anti-CD3- (1 *μ*g/ml) and anti-CD28- (2 *μ*g/ml) specific antibodies for 96 h. In order to assess the proliferation of CD4^+^ (a) and CD8^+^ (b) T cells, ^3^H thymidine (0.5 *μ*Ci/well) was applied for the last 16-18 h of culture. ^3^H thymidine uptake is presented. Data are the means ± SEM (*n* = 3 animals per genotype) and are pooled from three independent experiments (^∗∗^ *p* < 0.01 and ^∗^ *p* < 0.05; ns = not significant, two-way Anova/Bonferroni test). Expression of the indicated surface markers in polyclonally stimulated CD4^+^ and CD8^+^ T cells isolated from KSRP^+/+^ (WT) and KSRP^−/−^ (KO) mice was assessed by flow cytometry. One representative FACS analysis from two to three independent experiments is shown demonstrating CD4 (c) (APC-anti-CD4), CD8 (d) (eFluor450-anti-CD8), and CD25 (FITC-anti-CD25 mAb) expression. Data are the means+SEM (*n* = 2‐3 animals per genotype) and are pooled from 2 to 3 independent experiments (^∗∗^ *p* < 0.01; ns = not significant, unpaired *t*-test).

Next, CD4^+^ and CD8^+^ T cells were isolated from KSRP^−/−^ (KO) and wild-type (WT) mice and polyclonally stimulated as described in Materials and Methods to measure T cell proliferation. Our results indicate that CD4^+^ T cells from KSRP^−/−^ mice display a higher proliferation rate than the WT control (Figure 1(a)). In agreement, a higher frequency of KSRP^−/−^ CD4^+^ T cells expressed CD25 as compared with the WT control (Figure 1(c)). In contrast, no significant genotype-specific differences in CD8^+^ T cell proliferation and CD25 expression were detected (Figures 1(b) and 1(d)). Therefore, we focused on further analyses of KSRP^−/−^ CD4^+^ T cells only.

### 3.2. KSRP Changes the Cytokine Pattern of CD4^+^ T Cells

Next, we characterized the cytokine profile in supernatants of polyclonally stimulated CD4^+^ T cells by cytometric bead array analyses (CBA). Supernatants were obtained from CD4^+^ T cell cultures used for the proliferation assays described above.

**Figure 2:**
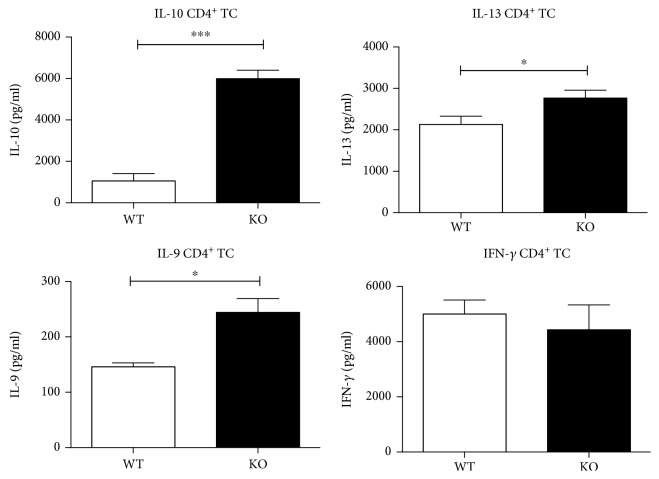
Effect of inactivation of the KSRP gene on T cell-mediated cytokine production. In supernatants of polyclonally stimulated CD4^+^ T cells isolated by magnetic bead separation from KSRP^+/+^ (WT) and KSRP^−/−^ (KO) mice, ΙL-10, IL-13, IL-9, and IFN-*γ* contents were measured by CBA. Shown data are the means+SEM (*n* = 3 animals per genotype) and are pooled from three independent experiments (^∗∗∗^ *p* < 0.001, ^∗∗^ *p* < 0.01, and ^∗^ *p* < 0.05; ns = not significant, unpaired *t*-test).

Our results demonstrate a sixfold increase in IL-10 and a twofold increase in IL-9 levels in CD4^+^ T cells isolated from KSRP^−/−^ mice compared to those isolated from the WT controls (Figure 2). A slight but significant increase was also detected for IL-13 production in KSRP^−/−^ CD4^+^ T cells, while IFN-*γ* levels were comparable (Figure 2). No significant changes in IL-17 levels were observed in these experiments (Sup.  Figure 2(a)).

**Figure 3:**
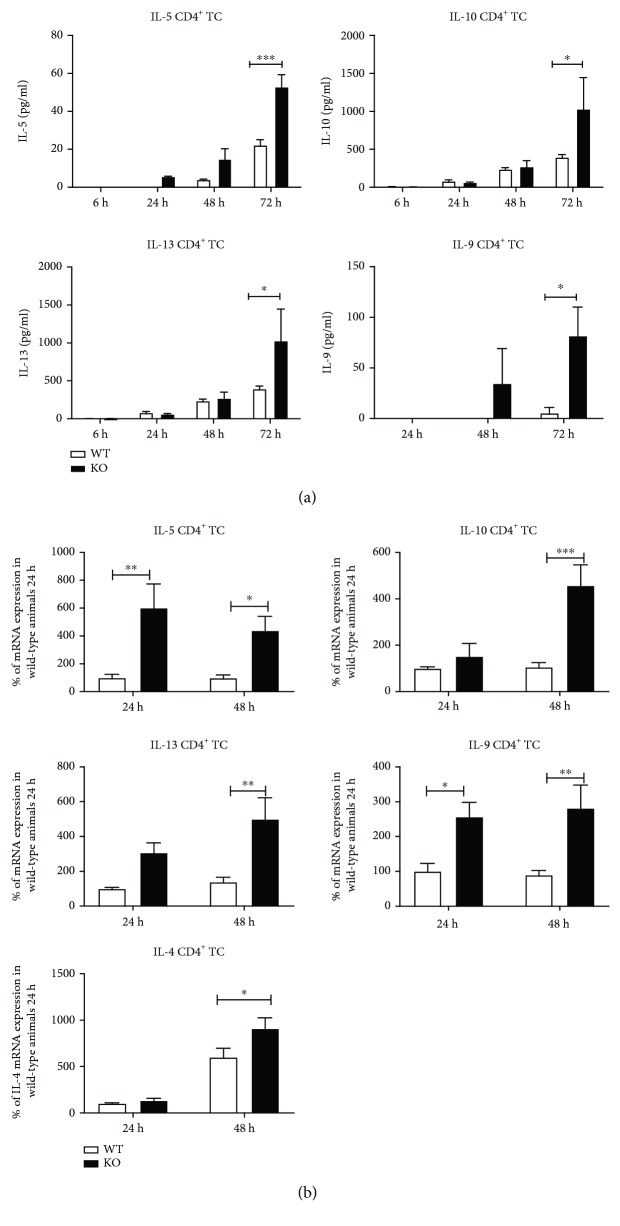
Time kinetics of T cell-mediated cytokine production. In supernatants from 6 h, 24 h, 48 h, and 72 h polyclonally stimulated CD4^+^ T cells isolated by magnetic bead separation from KSRP^+/+^ (WT) and KSRP^−/−^ (KO) mice, IL-5, ΙL-10, IL-13, and IL-9 contents were measured by CBA (a). Shown data are the means+SEM (*n* = 3‐4 animals per genotype) and are pooled from two technical replications (^∗∗∗^ *p* < 0.001 and ^∗^ *p* < 0.05; two-way Anova/multiple comparison test). (b) In CD4^+^ T cells from KSRP^+/+^ (WT) and KSRP^−/−^ (KO) mice used for CBA analyses, mRNA expression of IL-5, IL-10, IL-13, IL-9, and IL-4 was measured by qRT-PCR 24 h and 48 h after polyclonal stimulation of the cells. The mRNA expression of the different cytokines was normalized to Pol2a or 18S rRNA mRNA expression. The mRNA expression of KSRP^+/+^ mice polyclonally stimulated for 24 h was set to 100%. Shown data are the means+SEM (*n* = 3‐4 animals per genotype) and performed in duplicate (^∗∗∗^ *p* < 0.001, ^∗∗^ *p* < 0.01, and ^∗^ *p* < 0.05; two-way Anova/multiple comparison test).

The data described above were derived from supernatants obtained 80 h after polyclonal stimulation of T cells. As T cells may either consume or accumulate cytokines in the supernatant over time, this could affect our results. To resolve this problem, we performed time kinetic analysis of cytokine production in the supernatant of polyclonally stimulated CD4^+^ T cells. In these analyses, we also included IL-5 to evaluate whether the production of this prototypical Th2 cytokine, besides IL-13, is altered in CD4^+^ T cells derived from KSRP^−/−^ mice (KO). There was no cytokine production detected at all up to three hours after the onset of the polyclonal stimulation of CD4^+^ T cells. Therefore, in Figure 3(a) only results from 6 h to 72 h are presented. For all cytokines analyzed (IL-5, IL-9, IL-10, and IL-13), we detected an increased production over time. Significant differences between wild-type and knockout mice were detected 72 h after polyclonal stimulation of the T cells (Figure 3(a)). As KSRP primarily regulates mRNA expression, we checked whether the same tendency was observed on mRNA level. We used RNA isolated from CD4^+^ T cells 24 h and 48 h after polyclonal stimulation because at these time points remarkable cytokine expression was detected in CBA analyses. Indeed, we measured increased mRNA expression of IL-5, IL-9, IL-10, and IL-13 in CD4^+^ T cell RNA samples isolated from KSRP^−/−^ mice in comparison to the WT control group (Figure 3(b)). Alterations in mRNA expression levels were also time dependent and seem to precede changes in protein production. Altogether, these results support our data derived from CBA analyses and demonstrate KSRP-mediated regulation of IL-5, IL-9, IL-10, and IL-13 expression in CD4^+^ T cells. Therefore, it is tempting to speculate that the results described above are the consequence of KSRP-mediated modulation of mRNA expression.

T cells produce and consume IL-4 at the same time [22]. This might be the reason for the problems in detecting substantial IL-4 expression in supernatants of polyclonally stimulated T cells. Nevertheless, on the mRNA level we saw a significant increase of IL-4 mRNA expression in KSRP^−/−^ CD4^+^ T cells compared to wild-type controls (Figure 3(b)). This suggests that IL-4 production is inhibited by KSRP also. Taken together, these data imply that CD4^+^ T cells isolated from KSRP^−/−^ mice display an inherent Th2 bias as reflected by increased production of associated immune regulatory cytokines (IL-4, IL-5, IL-9, IL-10, and IL-13).

### 3.3. Cytokine Pattern in T Cells Derived from CAIA-Treated KSRP^−/−^ Mice

**Figure 4:**
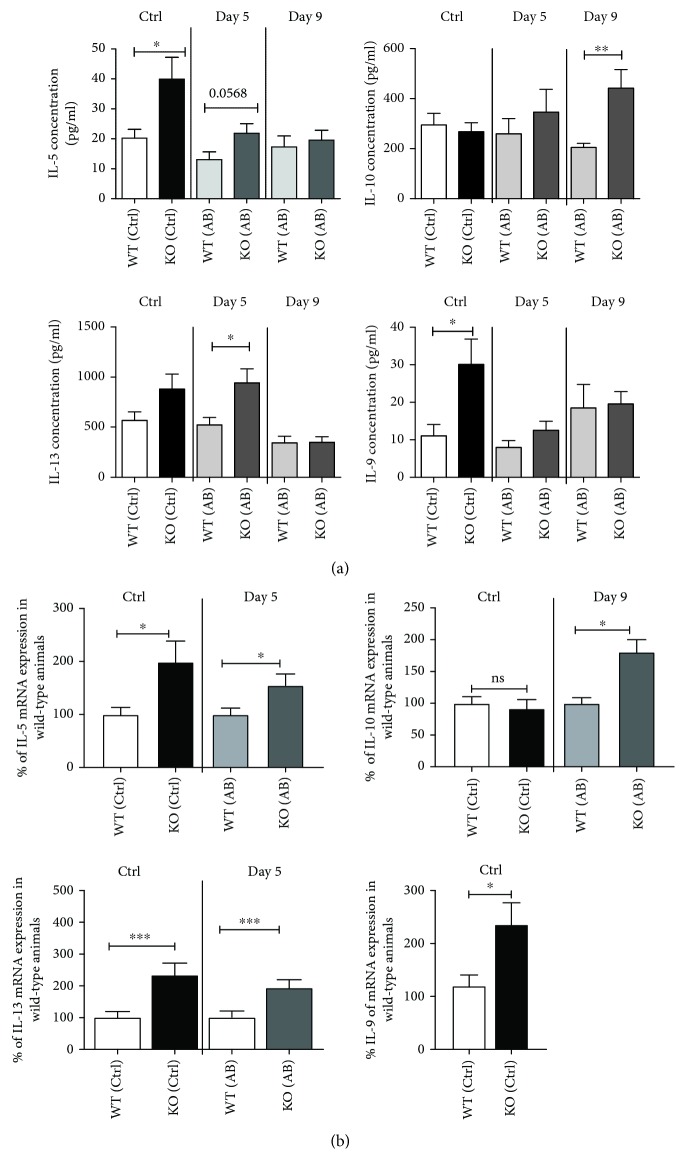
Effect of KSRP knockdown on T cell-mediated cytokine production in CAIA-treated mice. KSRP^+/+^ (WT) and KSRP^−/−^ (KO) mice were treated on day 0 with a cocktail of 5 collagen-II-specific mAbs (AB) or with PBS (Ctrl) as control. On day 3, the animals were treated with LPS (50 *μ*g/animal) or PBS as control. At day 5 and day 9, mice (*n* = 10‐15) of each treatment group were killed for subsequent analyses. Nylon wool-enriched T cells isolated from the spleens of KSRP^+/+^ (WT) and KSRP^−/−^ (KO) mice treated with (AB) or without (Ctrl) CII-specific mAbs were polyclonally stimulated with anti-CD3- (1 *μ*g/ml) and anti-CD28- (2 *μ*g/ml) specific antibodies for 96 h. IL-5, IL-10, IL-13, and IL-9 expression was measured in the supernatant of the cells using CBA (a). Data shown are the means+SEM (*n* = 10‐15 animals per genotype at days 5 and 9) and are pooled from four independent experiments (^∗∗^ *p* < 0.01 and ^∗^ *p* < 0.05; unpaired *t*-test). From a subset of these cells, RNA was isolated and mRNA expression of IL-5, IL-10, IL-13, and IL-9 was determined (b). The mRNA expression of the different cytokines was normalized to Pol2a mRNA expression. The mRNA expression of KSRP^+/+^ mice was set to 100%. Data shown are the means+SEM (*n* = 10‐15 animals per genotype at days 5 and 9) and are pooled from four independent experiments (^∗∗∗^ *p* < 0.001 and ^∗^ *p* < 0.05; ns=not significant, unpaired *t*-test).

Collagen antibody-induced arthritis is an established model to analyze the pathogenesis of rheumatoid arthritis in C57BL/6 mice [23]. As mentioned above, CAIA disease progression was attenuated in C57BL/6 KSRP^−/−^ mice, and as T cells were reported to modify CAIA disease progression [15], we were interested which changes occur in T cell populations in KSRP^−/−^ mice under proinflammatory conditions. In samples derived from CAIA-treated C57BL/6 KSRP^−/−^ mice [14], T cells were isolated from spleen homogenates at days 5 and 9 after intravenous application of the antibody cocktail. At day 5, the first clinical arthritis symptoms like swelling of the joints occur, whereas on day 9 the disease is in a steady state phase. So, we analyzed the impact of CAIA on T cell polarization at two different phases of the immune response in this disease model, within the effector phase at day 5 (d5) and during a more regulatory phase on day 9 (d9). Total T cells isolated from the spleens of CAIA-treated mice (AB) or PBS control (Ctrl) animals were polyclonally stimulated, and proliferation as well as cytokine contents were analyzed. Also, in these total splenic T cell pools we observed genotype-specific differences in T cell proliferation derived from PBS-treated control animals. Comparable to results obtained from KSRP^−/−^ CD4^+^ T cells (see Figure 1), we saw increased proliferation of splenic KSRP^−/−^ T cells (Sup.  Figure 3). We detected a significantly increased production of IL-5 and IL-9 in anti-CD3/CD28-stimulated splenic T cells isolated from PBS-treated KSRP^−/−^ animals compared to the WT control. In addition, there is strong evidence that the IL-13 concentration is also higher in KSRP^−/−^ T cells under control conditions (Sup.  Figure 4). At day 5 after CAIA induction, only IL-13 production was increased in T cells isolated from antibody-treated KSRP^−/−^ mice. No statistically significant differences in IL-5 and IL-9 expression were detected on days 5 and 9 after injection of the antibody cocktail. On day 9 after CAIA induction, we measured a robust increase in the dual Th2/Treg marker cytokine IL-10 in KSRP^−/−^ T cells compared to WT T cells. Furthermore, our data indicate an enhanced production of the Th2-inducing and Th2-associated cytokine IL-4 in PBS-treated control KSRP^−/−^ T cells (Sup.  Figure 5). In general, as shown in Figure 3, T cells isolated from KSRP^−/−^ mice after polyclonal stimulation displayed a Th2/Th9 bias which is partially retained in T cells derived from mice after the onset of CAIA (day 5). In the regulatory phase (day 9), expression of anti-inflammatory IL-10 was strongly induced in KSRP^−/−^ mice. No statistically significant differences in IL-17 expression were detected (Sup.  Figure 2(b)). We additionally analyzed mRNA expression of IL-5, IL-9, IL-10, and IL-13 in those samples which displayed significant changes in cytokine production. Analogous to changes in protein expression, significantly enhanced mRNA levels of IL-5 and IL-13 were also detected in splenic T cells of KSRP^−/−^ control animals and KSRP^−/−^ animals on d5 after the induction of CAIA compared to WT mice (Figure 4(b)). In agreement with CBA analyses, we saw enhanced IL-9 mRNA expression only in samples derived from KSRP^−/−^ mice without treatment (control) (Figure 4(b)). The IL-10 mRNA expression (Figure 4(b)) was regulated in the same way as the IL-10 cytokine production (Figure 4(a)). No genotype-specific differences in IL-10 mRNA expression were detected between PBS-treated WT and KSRP^−/−^ animals. Only in T cells isolated from KSRP^−/−^ mice at day 9 after CAIA induction was a significant increase of IL-10 mRNA observed. To sum up, in the CAIA model we observed elevated production of Th2-inducing and Th2-asssociated IL-4 by polyclonally stimulated T cells derived from KSRP^−/−^ control animals. In addition, we detected an upregulation of Th2-associated IL-5, IL-13, and IL-10 as well as of IL-9 on mRNA and protein level in T cells derived from KSRP^−/−^ mice in the course of CAIA progression. This suggests KSRP-mediated regulation of these cytokines.

### 3.4. KSRP Changes IFN-*γ* Expression in CD8^+^ T Cells and in T Cells Isolated from CAIA Mice

**Figure 5:**
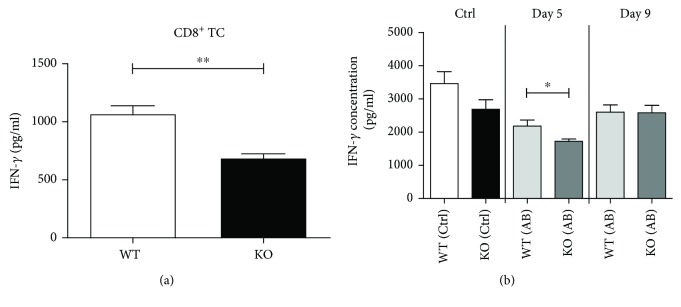
Effect of inactivation of the KSRP gene on IFN-*γ* production in T cells. Magnetic bead separation was used to isolate untouched CD8^+^ T cells from KSRP^+/+^ (WT) and KSRP^−/−^ (KO) mice. T cells were polyclonally stimulated with plate-bound anti-CD3- (1 *μ*g/ml) and anti-CD28- (2 *μ*g/ml) specific antibodies for 96 h. In supernatants of these cells, IFN-*γ* contents were measured by CBA (a). Shown data are the means+SEM (*n* = 3 animals per genotype) and are pooled from three independent experiments (^∗∗^ *p* < 0.01; unpaired *t*-test). KSRP^+/+^ (WT) and KSRP^−/−^ (KO) were treated on day 0 with a cocktail of 5 collagen-II-specific mAbs (AB) or with PBS (Ctrl) as control. On day 3, the animals were treated with LPS (50 *μ*g/animal) or PBS as control. At day 5 and day 9, mice (*n* = 10‐15) of each treatment group were killed for subsequent analyses. Nylon wool-enriched T cells isolated from the spleens of KSRP^+/+^ (WT) and KSRP^−/−^ (KO) mice treated with (AB) or without (Ctrl) CII-specific mAbs were polyclonally stimulated with anti-CD3- (1 *μ*g/ml) and anti-CD28- (2 *μ*g/ml) specific antibodies for 96 h. IFN-*γ* production of these cells was monitored by CBA (b). Data shown are the means+SEM (*n* = 10‐15 animals per genotype at days 5 and 9) and are pooled from four independent experiments. (^∗^ *p* < 0.05; unpaired *t*-test).

In supernatants of CD8^+^ T cells used to determine their proliferative capacity (Figure 1(b)), reduced Th1-associated IFN-*γ* production was detected in cells derived from KSRP^−/−^ mice compared to the WT controls (Figure 5(a)). A similar observation was made in splenic T cells isolated from CAIA mice. Quantification of cytokine production revealed a significantly reduced IFN-*γ* amount in supernatants of polyclonally stimulated T cells isolated from KSRP^−/−^ mice at day 5 after CAIA induction. At day nine after the application of the antibody cocktail, no genotype-dependent difference in levels of IFN-*γ* was observed. These results imply a KSRP-mediated regulation of IFN-*γ* expression in T cells.

### 3.5. Direct Binding of KSRP to the 3′-UTR of the IFN-*γ*, IL-10, and IL-9 mRNA

Next, we were interested whether the effects detected on cytokine production in T cells isolated from KSRP^−/−^ mice are a consequence of the direct interaction of KSRP with the 3′ untranslated region of the corresponding mRNAs, which should destabilize the message.

**Figure 6:**
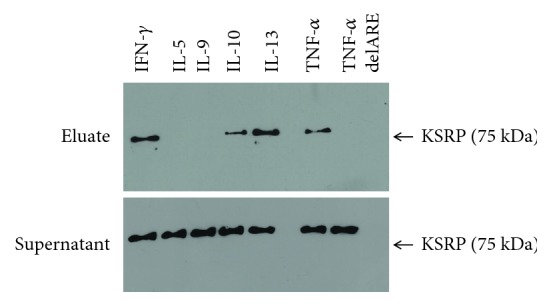
In vitro binding of KSRP to different 3′-UTRs. In vitro-transcribed biotinylated IFN-*γ*, IL-5, IL-10, IL-9, and TNF-*α* 3′-UTR or TNF-*α* 3′-UTR without ARE (delARE) RNAs were incubated with whole cell extracts from murine EL4 T cells. After several washing steps, the bound proteins were eluted with high salt (2 M KCl). The eluted proteins were separated in SDS-PAGE. KSRP was identified by western blotting using a specific anti-KSRP antibody. In the eluate fraction (eluate), binding of KSRP to the different 3′-UTRs is shown. The supernatant represents the amount of KSRP not bound to streptavidin-agarose in pull-down analyses. The blot is representative of three other blots showing similar results.

Therefore, we cloned the 3′-UTR of IFN-*γ*, IL-5, IL-9, IL-13, and IL-10 into the pCR-Script vector. The derived plasmids were used to generate biotinylated 3′-UTR RNA probes to perform pull-down analyses. It is known that the KSRP protein is ubiquitously expressed [24]. Therefore, we used the murine EL4 lymphoma T cell line as a source of the KSRP protein. The biotinylated RNA transcripts were incubated with EL4 cell extracts, and RNA-protein-complexes were isolated with streptavidin-agarose. To analyze KSRP binding to the in vitro-transcribed 3′-UTR constructs, western blot experiments were performed. The 3′-UTR of the TNF-*α* mRNA, a known KSRP target, was used as positive control, and a TNF-*α* 3′-UTR without ARE sequences served as a negative control. Our results demonstrate KSRP binding to the 3′-UTR of IFN-*γ*, IL-10, and IL-13 (Figure 6, eluate). In these experiments, no direct interaction of KSRP with the 3′-UTR of IL-5 and IL-9 was detectable. The supernatant fraction represents the amount of KSRP present in the protein extract used for pull-down analyses that does not bind to streptavidin-agarose beads (Figure 6, supernatant). Despite direct binding of KSRP to the 3′-UTR of IFN-*γ*, IL-10, and IL-13, no differences of IFN-*γ*, IL-10, or IL-13 mRNA decay in T cells derived from KSRP^−/−^ mice were detected compared to wild-type control cells (Sup.  Figure 6). These results argue against a direct effect of KSRP on IFN-*γ*, IL-10, or IL-13 mRNA stability.

**Figure 7:**
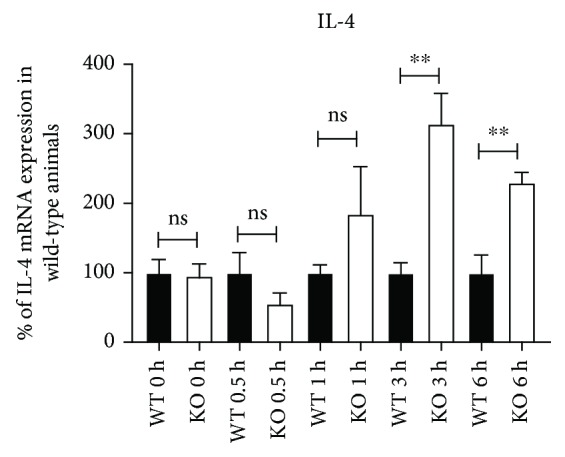
IL-4 mRNA stability in polyclonally stimulated T cells. CD4^+^ T cells from the spleens of KSRP^+/+^ (WT) and KSRP^−/−^ (KO) mice were isolated by magnetic bead separation and were polyclonally stimulated with CD3/CD28 antibodies for 24 h. Then, 25 *μ*g/ml 5,6-dichloro-1-*β*-D-ribofuranosylbenzimidazole (DRB) (Sigma-Aldrich, Deisenhofen, Germany) was added to examine the influence of KSRP on the stability of IL-4 mRNA. RNAs were prepared 0, 0.5, 1, 3, and 6 h thereafter. mRNA amount was determined by qRT-PCR by normalizing to 18S rRNA and GAPDH expression. The relative IL-4 mRNA amount at 0, 0.5, 1, 3, and 6 h DRB was set at 100% in wild-type cells. Data shown are the means+SEM of two analyses (^∗∗^ *p* < 0.01; ns=not significant from wild-type mice, unpaired *t*-test).

IL-4 is one of the most important inducers of Th2 polarization and specifically produced by Th2 cells [25]. Therefore, we analyzed IL-4 mRNA decay in polyclonally stimulated T cells derived from KSRP^−/−^ mice. Indeed, enhanced expression of IL-4 mRNA in KSRP^−/−^ CD4^+^ T cells was detected upon the addition of an inhibitor of RNA-polymerase II-dependent transcription (Figure 7 and Sup.  Figure 7). This result might explain the Th2 phenotype of KSRP^−/−^ CD4^+^ T cells.

### 3.6. Regulation of IL-2 Expression by KSRP

**Figure 8:**
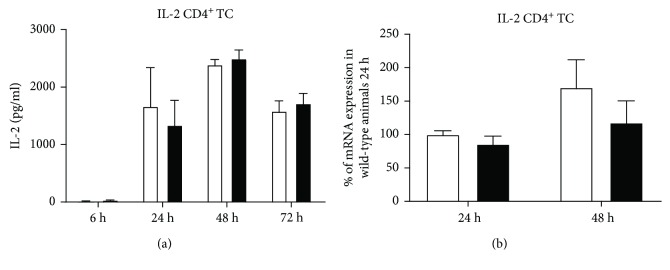
IL-2 expression in polyclonally stimulated T cells. In supernatants from 6 h, 24 h, 48 h, and 72 h polyclonally stimulated CD4^+^ T cells isolated by magnetic bead separation from KSRP^+/+^ (WT) and KSRP^−/−^ (KO) mice, IL-2 were measured by CBA (a). Shown data are the means+SEM (*n* = 3‐4 animals per genotype) and are pooled from two technical replications. In CD4^+^ T cells from KSRP^+/+^ (WT) and KSRP^−/−^ (KO) mice used for CBA analyses, mRNA expression of IL-2 was measured by qRT-PCR 24 h and 48 h after polyclonal stimulation of the cells (b). The mRNA expression of IL-2 was normalized to Pol2a mRNA expression. The mRNA expression of KSRP^+/+^ mice polyclonal stimulated for 24 h was set to 100%. Shown data are the means+SEM (*n* = 3‐4 animals per genotype) and are pooled from two technical replications.

In vitro experiments hint towards a KSRP-mediated regulation of IL-2 expression by decreasing its mRNA stability in HeLa cells [11]. Therefore, it was obvious to speculate that the increased proliferation of polyclonally stimulated KSRP^−/−^ CD4^+^ T cells (see Figure 1(a)) might be due to an elevated IL-2 expression in these cells. We analyzed IL-2 protein and mRNA expression in polyclonally stimulated CD4^+^ T cells. At none of the considered time points were significant differences in IL-2 expression detected in samples derived from KSRP^−/−^ mice compared to WT mice neither on the protein nor on mRNA level (Figures 8(a) and 8(b)). In accordance to pull-down analyses, no direct interaction of KSRP with IL-2 3′-UTR was observed (data not shown). So our data present no evidence for KSRP-mediated regulation of IL-2 expression in murine CD4^+^ T cells.

## 4. Discussion

KSRP is an RBP that has been described to negatively regulate the expression of several cytokines and chemokines by the destabilization of the mRNA. These results were based on experiments performed with established cell culture lines, such as DLD-1 or HeLa cells, or primary cell lines isolated from KSRP^−/−^ mice, such as embryonic fibroblasts or peritoneal cells [7, 20, 26, 27]. Therefore, it was expected that knockdown of the protein would enhance the expression of proinflammatory factors and this would aggravate inflammatory immune responses and diseases such as rheumatoid arthritis in vivo. But in C57BL/6 KSRP^−/−^ mice in a well-established arthritis model, the opposite effect occurs: the animals are protected against CAIA. Recently, we presented evidence that these differences could be attributed, at least in part, to reduced numbers of myeloid cells, especially of neutrophils, in KSRP^−/−^ mice [14]. In line with our data, a recent report of Zhao et al. demonstrates the importance of KSRP for monocytic and granulocytic differentiation [28]. Neutrophils are important effector cells in the CAIA model since their depletion reduces the severity of disease [29].

The results from the CAIA model indicate a much more complex role of KSRP in the regulation of immune response as expected. Our data might indicate that the number of KSRP target genes *in vivo* is greater than it could be estimated by the up to now published results. The results obtained from our experiments may indicate that, besides innate immune responses, KSRP is also involved in the regulation of adaptive immune mechanisms.

Indeed, for the first time we were able to present evidence that KSRP could modify adaptive immune responses as knockdown of the protein enhances the proliferative capacity of CD4^+^ T cells and induces an overall Th2 bias and increased IL-9 production. In CD8^+^ T cells, knockdown of KSRP expression diminished IFN-*γ* production.

First of all, we detected no difference in general T cell numbers and activation status, measured by CD25 expression, in KSRP^−/−^ mice compared to control mice (Suppl.  Table 1). This argues against an important role of KSRP in thymic T cell development as it has been described, for example, for the RBPs ZFP36L1 and ZFP36L2. These two RBPs are important for proper B cell as well T cell development, due to their ability to control cell cycle progression in lymphopoiesis [30, 31]. In addition, their capacity to regulate Notch-1 expression also contributes to thymocyte development [32]. Nevertheless, we detected the enhanced proliferation of CD3/CD28-activated KSRP^−/−^ CD4^+^ splenic T cells and increased expression of the Th2 cytokines IL-5 and IL-13, as well as of IL-9 and IL-10 compared to control cells (Figures 1 and 2). It is well known that IL-2 is important for T cell proliferation. Besides transcriptional mechanisms, posttranscriptional regulation of mRNA stability is also involved in the control of IL-2 expression. ARE located in the IL-2 3′-UTR confers transcript instability, and therefore, it is not surprising that RBPs are involved in the regulation of IL-2 expression. In polyclonally activated T cells, direct interaction of the mRNA-destabilizing RBP tristetraprolin (TTP) with the IL-2 3′-UTR was detected. Accordingly, IL-2 mRNA and protein expression was increased in polyclonally stimulated T cells derived from TTP^−/−^ mice compared to wild-type control animals, indicating an important negative regulatory function of TTP on IL-2 gene expression [33]. Also, KSRP has been described to negatively regulate IL-2 expression by decreasing its mRNA stability in HeLa cells [11, 34]. Therefore, increased proliferation of polyclonally stimulated KSRP^−/−^ CD4^+^ T cells could be due to elevated IL-2 expression in these cells. We have checked this hypothesis, but we were not able to detect any difference in IL-2 production neither on mRNA nor on protein level between KSRP^+/+^ and KSRP^−/−^ mice in polyclonally stimulated CD4^+^ T cells (see Figure 8). One reason for this unexpected finding could be that CD4^+^ T cells from KSRP^−/−^ mice might consume more IL-2 than their wild-type counterparts due to their higher proliferation capacity, and therefore, no difference in IL-2 production was detectable in our experiments. In our opinion, the data from mRNA expression analyses argues against this hypothesis. At any time point analyzed, we were not able to detect a difference in IL-2 mRNA expression between KSRP^+/+^ and KSRP^−/−^ mice. In pull-down analyses, we detected no direct interaction of KSRP with the IL-2 3′-UTR. This also hints towards the direction that KSRP does not affect the regulation of IL-2 expression in T cells (data not shown). The discrepancies of our data to the data published by Gherzi et al. [11] might be due to the reason that the results of these authors were obtained from in vitro experiments using IL-2 3′-UTR reporter gene constructs either in HeLa cells or with Jurkat cell extracts, whereas our data were derived from primary T cells isolated from KSRP^−/−^ mice. Keep in mind that TTP is also involved in the regulation of IL-2 mRNA expression in T cells; this effect may override KSRP-mediated regulation mechanisms in vivo. This could explain why we were not able to detect KSRP-mediated IL-2 regulation in splenic CD4^+^ T cells. A conceivable possibility for enhanced proliferation and skewing towards a Th2 cell phenotype in KSRP^−/−^ CD4^+^ T cells may be altered activation of the canonical Wnt/*β*-catenin/TCF-1 pathway. Wnt/*β*-catenin/TCF-1 signaling is involved in T cell differentiation processes [35], and it has been described that KSRP probably negatively regulates Wnt/*β*-catenin signaling via an interaction with the Dishevelled 3 protein [36].

Furthermore, enhanced CD25 expression detected in CD3/CD28-activated CD4^+^ T cells derived from KSRP^−/−^ mice (Figure 1(c)) is most probably not the consequence of a direct KSRP effect on CD25 mRNA expression since we saw no differences in CD25 mRNA expression in polyclonally stimulated T cells of KSRP^−/−^ mice compared to KSRP^+/+^ mice (Sup.  Figure 8).

A similar prevalence of a more regulatory immune response was detected in polyclonally stimulated T cells isolated from the spleens of CAIA-treated KSRP^−/−^ mice. In untreated control mice and on day 5 after antibody treatment, in the effector phase of the disease, increased production of prototypical Th2 cytokines and Th9-associated IL-9 was detected in T cells isolated from KSRP^−/−^ mice (see Figure 4). These genotype-specific differences seem to be equalized in the course of the disease, because in T cells isolated on day nine after disease induction (regulatory phase) no obvious differences in cytokine production was detected. One exception was made by IL-10 whose expression was significantly enhanced in polyclonally stimulated T cells isolated from KSRP^−/−^ mice on day nine after CAIA induction. All cytokines differentially expressed in KSRP^−/−^ T cells are potent anti arthritic cytokines, and their increased expression has been connected with resolution of the disease [37]. As we analyzed effector and steady state phases of CAIA, this might explain the unchanged expression of IL-5, IL-9, IL-10, and IL-13 in T cells derived from KSRP^+/+^ mice during CAIA disease progression. On the other hand, the obvious intrinsic property of KSRP^−/−^ T cells to produce these anti arthritic cytokines upon activation in combination with the postulated disease-modifying role of T cells in the CAIA model [15] may contribute in combination with reduced myeloid cell number [14] to the attenuated disease phenotype in KSRP^−/−^ mice. It is well known that IFN-*γ* production by T cells is important for the onset and regulation of Th1 immune responses and macrophage activation [38]. The reduced amount of IFN-*γ* detected in polyclonally stimulated KSRP^−/−^ CD8^+^ T cells might be another parameter that impedes cellular immune response in arthritis development in CAIA-treated animals. In summary, these data implicate that knockdown of KSRP protein expression results in a Th2/Th9 bias of T cells isolated in the effector phase of CAIA, whereas T cells isolated in the steady state of the disease seem to be characterized by elevated IL-10 production in the knockout animals. These intrinsic T cell properties might contribute together with other factors [14] to the delayed onset and development of a prototypically Th1-dominated autoimmune disease, such as rheumatoid arthritis in KSRP^−/−^ mice.

All cytokine mRNAs whose expression is differentially regulated by KSRP (Figures 2 and 4) contain ARE in their 3′-UTR; however, not for all, ARE-mediated mRNA decay mechanisms have been described and none of them was identified to be a direct target of KSRP-mediated mRNA decay so far. Whereas the responsiveness of IL-10 and IL-13 mRNA for rapid degradation by ARE-binding RBPs (RBP TTP, AUF1, and HuR) [39–43] has been documented, much less is known about the mechanisms of the posttranscriptional regulation of IL-5 and IL-9 mRNA expression [44–46]. In line with that, we detected the direct binding of KSRP to the IL-10 and IL-13 3′-UTR but not to the IL-5 and IL-9 3′-UTR (Figure 6). One report already demonstrated a direct interaction of KSRP with the IL-10 3′-UTR in human HeLa cells [25], but it is unknown whether this alters IL-10 mRNA decay. Data from experiments using an inhibitor of RNA-polymerase IIA-dependent transcription to determine IFN-*γ*, IL-10, and IL-13 mRNA decay in CD4^+^ T cells derived from KSRP^−/−^ mice reveal only limited evidence for a direct effect of KSRP on mRNA decay (Sup.  Figure 6). Therefore, it remains puzzling to explain the effect of KSRP on cytokine expression in CD4^+^ T cells, because the results do not fit with the hypothesis of KSRP as a mRNA-destabilizing factor. Unfortunately, at the moment we are not able to provide molecular mechanisms on how KSRP may regulate IL-5, IL-9, IL-10, IL-13, or IFN-*γ* mRNA expression via direct or indirect mechanisms. KSRP is a multifunctional protein involved in different molecular processes regulating gene expression, such as miRNA maturation, interaction with long noncoding RNAs, transcription, or modulation of intracellular signaling pathways (Wnt signaling) [9, 10, 36, 47]. Whether either of these mechanisms is involved in KSRP-mediated changes of IL-5, IL-9, IL-10, IL-13, or IFN-*γ* mRNA and protein expression remains to be elucidated. However, since we had observed the elevated production of Th2-associated cytokines, we also assessed the expression of IL-4 which on one hand is the master regulator of Th2 polarization and on the other hand constitutes the prototypic Th2-associated cytokine [25]. As outlined above, the detection of IL-4 in T cell culture supernatants is complicated by the fact that T cells may considerably deplete this cytokine due to expression of the IL-4 receptor [22]. We could show that polyclonally stimulated KSRP^−/−^ CD4^+^ T cells expressed IL-4 on the mRNA (Figure 3(b)) and protein (Sup.  Figure 5) level at a higher extent than the corresponding WT control which may be explained by a longer mRNA half-life (Figure 7). Therefore, our data suggest that KSRP is a negative regulator of IL-4 expression and therefore dampens Th2 polarization.

Interestingly, the RNA-BP HuR counteracts KSRP in this regard as it posttranscriptionally enhances the expression of IL-4, favoring a Th2 polarization [43, 48]. We and others have shown that HuR and KSRP often compete with each other for binding to same target mRNAs and regulate their stability in an opposite way [26, 43]. Diminished KSRP expression might enhance HuR effects in polyclonally stimulated KSRP^−/−^ CD4^+^ T cells, and this may account as well for the observed Th2 skewing in our experiments. The reduced expression of IFN-*γ* detected in KSRP^−/−^ CD8^+^ T cells cannot be explained by direct KSRP-mediated mRNA destabilization. It seems more likely that the effects on IFN-*γ* expression detected in our experiments are due to KSRP-modulated changes in upstream signaling pathways such as IL-4 signaling which are responsible for Th2 T cell phenotype polarization.

In summary, our data are inconsistent with the hypothesis that a direct effect of KSRP on mRNA stability is responsible for the differential expression of IL-5, IL-9, IL-10, IL-13, and IFN-*γ* in T cells. Most likely, our data indicate a central role of KSRP in the regulation of IL-4 expression, which may explain the Th2 bias of KSRP^−/−^ CD4^+^ T cells. To support this hypothesis and to uncover the molecular mechanisms responsible for the Th2 cytokine shift in KSRP^−/−^ CD4^+^ T cells, RNA-seq analyses will be performed.

## 5. Conclusion

In summary, our data show for the first time that KSRP is able to modulate IL-4, IL-5, IL-10, IL-13, IL-9, and IFN-*γ* cytokine production in T cells and enhance CD4^+^ T cell proliferation upon polyclonal stimulation. These results implicate that the loss of the KSRP protein in mice induces a Th2/Th9 bias and thus may attenuate the development of Th1 immune responses.

## Figures and Tables

**Table 1:** Oligonucleotides used for the amplification of the different 3′-UTR sequences.

Oligonucleotide	Sense	Antisense
IL-9	TGAAATATTATTTATCC	TTTATTTCAAAATAAAGAC
IL-10	CATCAGGGTGGCGACTCTATAG	TGTGAATAAGATACATTTATTTATTCAAAATTAAAATC
IFN-*γ*	TGGTTGTCCTGCCTGCAATATTTG	GTTGTGAACTTACACTTTATTC
IL-5	CTAAACTGGTTTGTTGCAGC	ACAGTTGTCTATTTTTGTTTTATTAGAAC
IL-13	ACTTCGAAAGCATCATTATTTGC	TCTGTCACCAACTTTATTTCTGG
IL-2	TAATTAAGTGCTTCCCACTTAAAAC	CAAATATCCAGGCTTGTTTATATTTATC

**Table 2:** Oligonucleotides used for quantitaive real time RT-PCR experiments.

Oligonucleotide	Sense	Antisense
Murine IL-2	CCTGAGCAGGATGGAGAATTACA	TCCAGAACATGCCGCAGAG
Murine IL-4	ACAGGAGAAGGGACGCCAT	GAAGCCCTACAGACGAGCTCA
Murine IL-5	GCTCTGTTGACAAGCAATGAGACG	CTCTTGCAGGTAATCCAGGAACTG
Murine IL-10	TGAAAATAAGAGCAAGGCAGTG	TCATTCATGGCCTTGTAGACAC
Murine IL-13	AGACCAGACTCCCCTGTGCA	TGGGTCCTGTAGATGGCATTG
Murine Pol2a	ACCACGTCCAATGATATTGTGGAG	ATGTCATAGTGTCACACAGGAGCG
Murine IFN-*γ*	TCAAGTGGCATAGATGTGGAAGAA	TGGCTCTGCAGGATTTTCATG
Murine IL-9	CTGATGATTGTACCACACCGTGC	GCCTTTGCATCTCTGTCTTCTGG
Murine CD25	GCAGAGAATTTCATCCAGTTCC	TTGCATTCACAGTTTAGGATGG
Murine GAPDH	TTCACCACCATGGAGAAG GC	GGCATGGACTGTGGTCAT GA
18S rRNA	CGGCTACCACATCCAAGGAA	GCTGGAATTACCGCGGCT

## Data Availability

The data used to support the findings of this study are available from the corresponding author upon request.

## Abbreviations

3′-UTR: 3′ untranslated region
AI: Arthritis index
ARE: AU-rich element
ARE-BP: ARE-binding protein
CAIA: Collagen antibody-induced arthritis
CBA: Cytometric bead array
CII: Collagen type II
FACS: Fluorescent-activated cell sorting
FuBP2: Far upstream sequence-binding protein 2
KSRP: KH-type splicing regulatory protein
RA: Rheumatoid arthritis
RBP: RNA-binding protein
TTP: Tristetraprolin.

## Supplementary Materials

Supplementary Materials Figure S1: the knockdown of KSRP protein in CD4^+^ T cells derived from KSRP^−/−^ mice was demonstrated by western blot experiments. Supplemental Table 1: in spleen cells, no genotype-specific differences in the total number of CD4^+^ and CD8^+^ T cells were detected in FACS analyses. Figure S2: no significant differences in IL-17 expression were detected in CD4^+^ T cells. Figure S3: upon anti-CD3/CD28 stimulation, enhanced proliferation of total T cells isolated from the spleen of KSRP^−/−^ mice was detected compared to wild-type mice. Figure S4: effect of KSRP knockdown on T cell-mediated IL-13 production in CAIA-treated mice. Figure S5: cytometric bead array analyses demonstrated enhanced IL-4 expression in KSRP^−/−^ CD4^+^ T cells. Figure S6 and S7: mRNA decay analyses of IFN-*γ*, IL-10, IL-13, and IL-4 in T cells. Figure S8: in qRT-PCR analyses, no effect of KSRP on CD25 mRNA expression was detected in polyclonally stimulated CD4^+^ T cells.
