# Supplementary material for: The RNA-Binding Protein KSRP Modulates Cytokine Expression of CD4+ T Cells
Source: J Immunol Res. 2019 Aug 14;2019:4726532. doi: 10.1155/2019/4726532 (PMC6714327; doi:10.1155/2019/4726532)
Supplement: Supplementary Materials — Figure S1: the knockdown of KSRP protein in CD4+ T cells derived from KSRP−/− mice was demonstrated by western blot experiments. Supplemental Table 1: in spleen cells, no genotype-specific differences in the total number of CD4+ and CD8+ T cells were detected in FACS analyses. Figure S2: no significant differences in IL-17 expression were detected in CD4+ T cells. Figure S3: upon anti-CD3/CD28 stimulation, enhanced proliferation of total T cells isolated from the spleen of KSRP−/− mice was detected compared to wild-type mice. Figure S4: effect of KSRP knockdown on T cell-mediated IL-13 production in CAIA-treated mice. Figure S5: cytometric bead array analyses demonstrated enhanced IL-4 expression in KSRP−/− CD4+ T cells. Figure S6 and S7: mRNA decay analyses of IFN-γ, IL-10, IL-13, and IL-4 in T cells. Figure S8: in qRT-PCR analyses, no effect of KSRP on CD25 mRNA expression was detected in polyclonally stimulated CD4+ T cells. [file 4726532.f1.pdf]

Supplemental Data

1) Knockdown of KSRP protein in CD4+ T cells

We performed western blot analyses to demonstrate loss of KSRP protein expression in CD4+ T cells derived from KSRP-/- (**KO**) mice. Therefore, CD4+ T cells from KSRP+/+ (**WT**) and KSRP-/- (**KO**) mice were isolated by magnetic bead separation as described in the method section of the main manuscript. To obtain protein extracts for western blot experiments, CD4+ T cells were lysed in RIPA buffer (50 mM Tris-HCl [pH 7.4], 150 mM NaCl, 2mM EDTA, 10% glycerol, 1% NP40, 1x complete EDTA-free protease and phosphatase inhibitor cocktail) using an ultrasonic homogenizer. Protein concentration was determined using Protein Assay Dye Reagent (Bio-Rad, München, Germany) as described by the manufacturer and 100 µg of CD4+ T cell extract were separated by SDS-gel electrophoresis. KSRP protein expression was analyzed with a specific anti-KSRP antibody (abcam, ab229660). For normalization, the expression of GAPDH was analyzed in parallel using a specific anti-GAPDH antibody (Santa Cruz Biotechnology, 32233, Dallas, USA). The immunoreactive proteins on the blot were visualized by the enhanced chemiluminescence detection system (Thermo Fisher Scientific, Darmstadt, Germany).

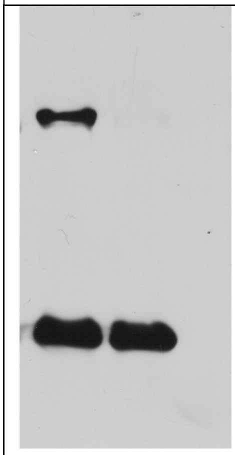

Sup. Figure 1: ***Knockdown of KSRP protein in CD4+T cells***

Expression of KSRP in CD4+ T cells from KSRP+/+ (**WT**) and KSRP-/- (**KO**) mice, isolated by magnetic bead separation, was analyzed by western blot experiments using a specific anti-KSRP antibody. For normalization, the expression of GAPDH was analyzed in parallel using a specific anti-GAPDH antibody. The blot is representative of two blot showing similar results.

2) T cell numbers in $KSRP^{-/-}$ mice

To figure out whether KSRP deficiency effect the T cell compartment we isolated total spleen cells from $KSRP^{+/+}$ (**WT**) and $KSRP^{-/-}$ (**KO**) mice. Spleens were removed and erythrocytes within the spleen cell suspension were lyzed by incubation of cells for 1 min in hypotonic buffer (155 mM NH_4Cl, 10 nM $KHCO_3$, 100 µM EDTA-disodium, pH 7.4). All cells were incubated with PE-anti-CD3, APC-anti-CD4, eFluor450-anti-CD8 mAbs and FITC-anti-CD25 mAb. Cells were analyzed using a FACS Canto II. Data are shown are the mean MFI ± SEM (n=2-3 animals per genotype) and are pooled from 2-3 independent experiments. (ns = not significant, unpaired T-test).

	**KSRP WT**	**$KSRP^{-/-}$**	
**$CD\ 4^+$**	13348.67 ± 4403.45	12799.33 ± 4655.84	ns
**$CD\ 8^+$**	1957.00 ± 117.95	1837.00 ± 49.15	ns

Sup. Table 1: ***Mean Fluorescence Intensity (MFI) of $CD4^+$ and $CD8^+$ T cells in $KSRP^{-/-}$ mice***

3) IL-17 production in KSRP$^{-/-}$ T cell

a)

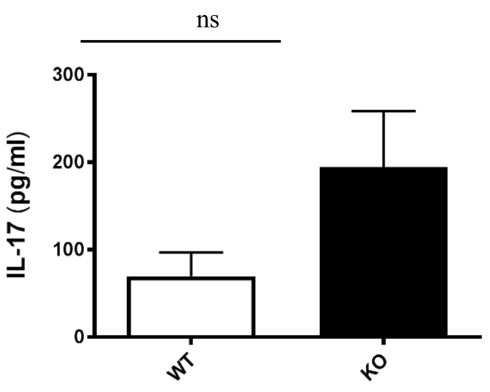

b)

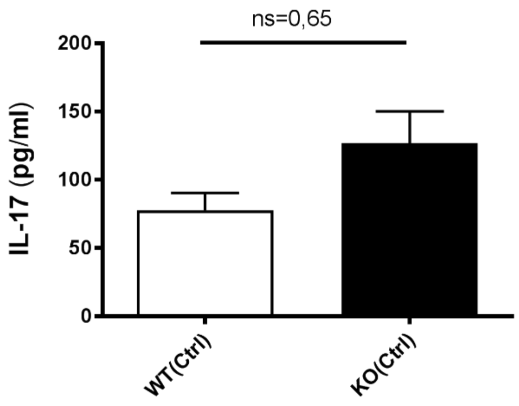

**CD4^{+} T cells**	**CAIA T cells**

Sup. Figure 2*: Effect of inactivation of the KSRP gene on T cell mediated IL-17 production*

(a) In supernatants of polyclonally stimulated CD4^{+} T cells isolated by magnetic bead separation from KSRP$^{+/+}$ (**WT**) and KSRP$^{-/-}$ (**KO**) mice the IL-17 content was measured by CBA. Shown data are the mean + SEM (n=3 animals per genotype). No statistically significant differences were detected. (b) Nylon wool-enriched T cells isolated from spleens of KSRP$^{+/+}$ (**WT**) and KSRP$^{-/-}$ (**KO**) mice treated without CII-specific mAbs (**Ctrl**) were polyclonally stimulated with anti-CD3 (1 µg/ml) and anti-CD28 (2 µg/ml) specific antibodies for 96 h. IL-17 expression was measured in the supernatant of the cells using CBA. Data are shown are the mean IL-17 production in pg/ml + SEM (n=10-15 animals per genotype and are pooled from four independent experiments. (ns. = not significant different from wildtype mice; unpaired T-test).

4) Proliferation of total splenic T cells

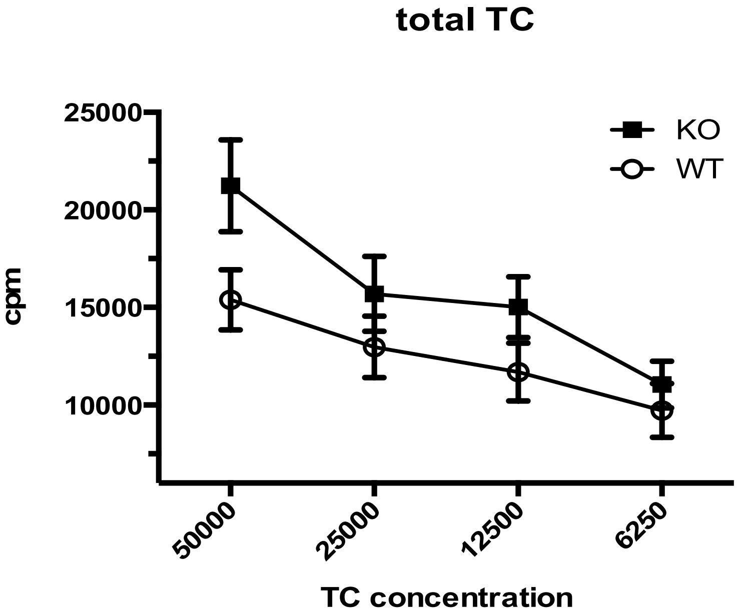

Sup. Figure 3: ***Proliferation of total T cells isolated from spleen***

Inactivation of the KSRP gene enhances proliferation of polyclonally stimulated T cells

T cells were isolated from spleen cells of $KSRP^{+/+}$ (**WT**) and $KSRP^{-/-}$ (**KO**) mice via nylon wool enrichment and were polyclonally stimulated with anti-CD3 (1 µg/ml) and anti-CD28 (2 µg/ml) specific antibodies for 96h. In order to assess the proliferation of T cells, ^{3}H thymidine (0.5 µCi/well) was applied for the last 16-18h of culture incubation. Total ^{3}H thymidine uptake is presented. Data are shown are the mean ± SEM (n=20-25 animals per genotype) and are pooled from five independent experiments.

5) IL-13 production in $KSRP^{-/-}$ T cells

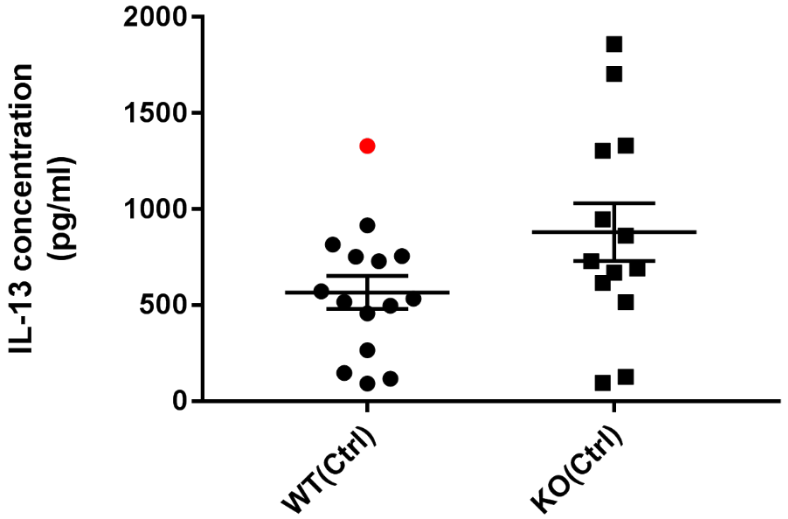

Sup. Figure 4*: Effect of KSRP knockdown on T cell mediated IL-13 cytokine production in CAIA-treated mice*

$KSRP^{+/+}$ (**WT**) and $KSRP^{-/-}$ (**KO**) were treated on day 0 with a cocktail of 5 different collagen-II-specific mAbs (**AB**) or with PBS (**Ctrl**) as control. On day 3 the animals were treated with LPS (50 µg/animal) or PBS as control. At day 5 and day 9 mice (n = 10-15) of each treatment group were killed for subsequent analyses. Nylon wool-enriched T cells isolated from spleens of $KSRP^{+/+}$ (**WT**) and $KSRP^{-/-}$ (**KO**) mice treated with (**AB**) or without (**Ctrl**) CII-specific mAbs were polyclonally stimulated with anti-CD3 (1 µg/ml) and anti-CD28 (2 µg/ml) specific antibodies for 96 h. IL-13 expression was measured in the supernatant of the cells using CBA. Data shown are the mean ± SEM (n=10-15 animals per genotype) and are pooled from four independent experiments. Without red marked value statistical difference (* = $p < 0.05$ from wildtype mice, unpaired T-test).

6) IL-4 production in KSRP$^{-/-}$ T cells

a)

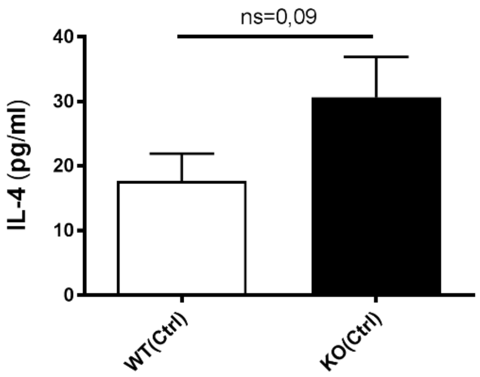

total IL-4 production

b)

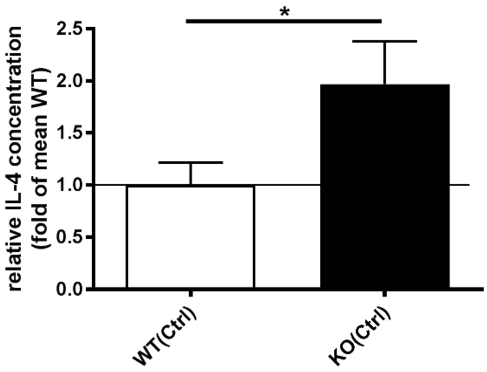

IL-4 production normalized to wild type control animals

Sup. Figure 5: ***Effect of KSRP knockdown on T cell mediated IL-4 production in control mice of CAIA experiments***

Nylon wool-enriched T cells isolated from spleens of KSRP$^{+/+}$ (**WT**) and KSRP$^{-/-}$ (**KO**) mice treated without (**Ctrl**) CII-specific mAbs were polyclonally stimulated with anti-CD3 (1 µg/ml) and anti-CD28 (2 µg/ml) specific antibodies for 96 h. IL-4 expression was measured in the supernatant of the cells using CBA (**a**). Data are shown are the mean IL-4 production in pg/ml + SEM (n=10-15 animals per genotype and are pooled from four independent experiments. (ns. = not significantly different from wildtype mice; unpaired T-test). (**b**). To normalize for the inter-individual cytokine production, IL-4 expression of KSRP$^{-/-}$ (**KO**) T cells was normalized to that of KSRP$^{+/+}$ (**WT**) T cells. The IL-4 expression of KSRP$^{+/+}$ T cells was set to 1. Data are shown are the mean + SEM (n=10-15 animals per genotype and are pooled from four independent experiments. (* = $p < 0.05$ from wildtype mice, unpaired T-test).

7) mRNA decay analyses in KSRP-/- T cells

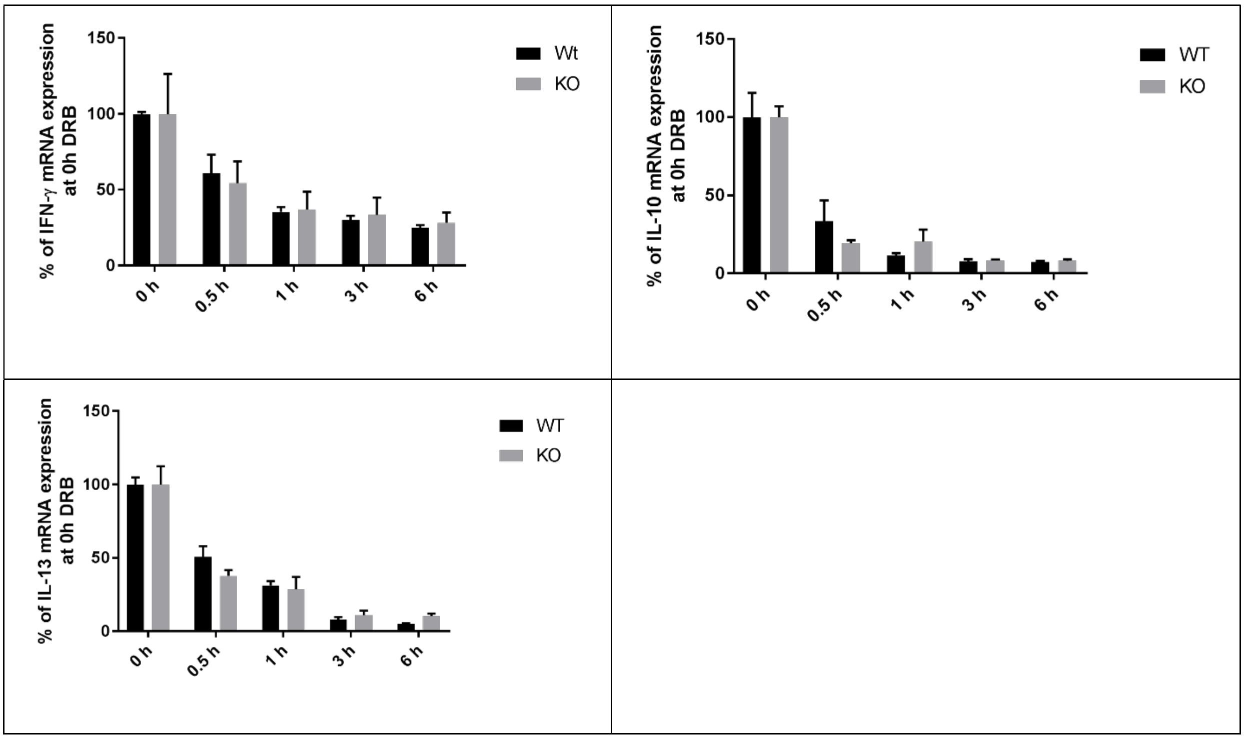

Sup. Figure 6: ***mRNA decay analyses***

CD4+ T cells from spleens of KSRP+/+ (**WT**) and KSRP-/- (**KO**) mice were isolated by magnetic bead separation, and were polyclonally stimulated with CD3/CD28 antibodies for 24h. Then 25 µg/ml 6-dichloro-1-ribofuranosylbenzimidazole (DRB) (Sigma, Deisenhofen, Germany) was added to examine the influence of KSRP on the stability of IFN-γ, IL-10 or IL-13 mRNA. RNAs were prepared 0, 0.5, 1, 3 and 6 h thereafter. mRNA amount was determined by qRT-PCR by normalizing to 18 S rRNA and GAPDH expression. The relative mRNA amount at 0 h DRB was set at 100% for each genotype. Data shown are the means + SEM of two analyses.

8) IL-4 mRNA decay in KSRP$^{-/-}$ T cells

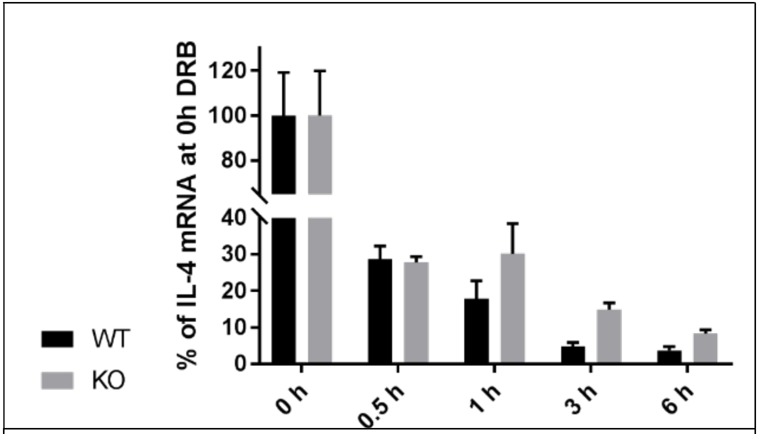

IL-4 mRNA expression normalized to each genotype

Sup. Figure 7: ***IL-4 mRNA stability analyses***

CD4$^+$ T cells from spleens of KSRP$^{+/+}$ (**WT**) and KSRP$^{-/-}$ (**KO**) mice were isolated by magnetic bead separation, and were polyclonally stimulated with CD3/CD28 antibodies for 24h. Then 25 µg/ml 6-dichloro-1-ribofuranosylbenzimidazole (DRB) (Sigma, Deisenhofen, Germany) was added to examine the influence of KSRP on the stability of IL-4 mRNA. RNAs were prepared 0, 0.5, 1, 3 and 6 h thereafter. mRNA amount was determined by qRT-PCR by normalizing to 18 S rRNA and GAPDH expression. The relative mRNA amount at 0 h DRB was set at 100% for each genotype.

9) CD25 mRNA expression KSRP$^{-/-}$ T cells

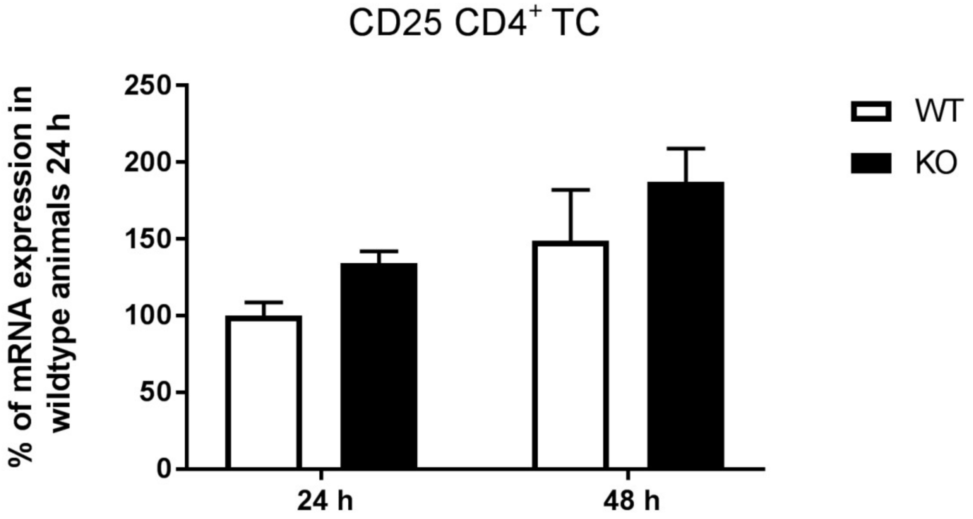

Sup. Figure 8: ***CD25 mRNA expression in CD4^{+} T cells of KSRP$^{-/-}$ mice***

In CD4^{+} T cells from KSRP$^{+/+}$ (**WT**) and KSRP$^{-/-}$ (**KO**) mice mRNA expression of CD 25 measured by qRT-PCR 24h and 48h after polyclonal stimulation of the cells. The mRNA expression was normalized to Pol2a mRNA expression. The mRNA expression of KSRP$^{+/+}$ mice polyclonal stimulated for 24h was set to 100%. Shown data are the mean + SEM (n=3-4 animals per genotype) and are pooled from two technical replications.
